# An Overview of In Vitro Release Methods for Long-Acting Injectable Products Based on PLGA

**DOI:** 10.3390/mps9030087

**Published:** 2026-06-01

**Authors:** Maja Lusina Kregar, Iva Krtalić, Ivana Šagud

**Affiliations:** 1 HALMED, Agency for Medicinal Products and Medical Devices of Croatia, Ksaverska Cesta 4, 10000 Zagreb, Croatia; maja.lusinakregar@halmed.hr; 2 Faculty of Medicine, University of Rijeka, 51000 Rijeka, Croatia; 3 Research and Development, PLIVA Croatia Ltd., Teva, Prilaz Baruna Filipovića 25, 10000 Zagreb, Croatia; iva.krtalic@pliva.com; 4 Faculty of Biotechnology and Drug Development, University of Rijeka, 51000 Rijeka, Croatia

**Keywords:** long-acting injectables, in vitro release test, PLGA, drug delivery systems, biorelevance

## Abstract

Long-acting injectables (LAIs) are widely used for chronic conditions such as schizophrenia, opioid use disorder, and HIV. Their prolonged efficacy improves adherence and reduces dosing frequency. Among these systems, poly(lactide-co-glycolide) (PLGA)-based formulations are commonly used to deliver drugs ranging from small molecules to peptides and proteins. In vitro release (IVR) tests play a critical role in evaluating drug product performance for both immediate- and prolonged-release dosage forms. However, there is a lack of standardized compendial IVR methods for the assessment of LAIs. This lack impedes the development of new drug products in this area and also complicates their regulatory approval process. Considering the complexity of drug release mechanisms and the diversity of various formulation design approaches, it is not possible to devise a universal IVR method that is applicable to all LAI products. The in vitro release test applied for quality control should be simple, robust, reproducible, and discriminatory. On the other hand, more complex biorelevant media and methods are often used during development to better reflect physiological conditions. This article provides a comprehensive review of compendial and non-compendial methods used for in vitro release testing of PLGA-based LAIs (microspheres and in situ forming implants), with the goal of aiding the development and standardization of future methodologies.

## 1. Introduction

Long-acting injectables (LAIs) have emerged as valuable drug delivery systems because they provide sustained and consistent drug exposure over extended periods, thereby reducing the dosing burden associated with long-term oral therapy and improving adherence [1]. This advantage is particularly beneficial in chronic conditions where consistent therapeutic coverage is essential, including schizophrenia, HIV treatment and prevention, contraception, oncology, hormonal disorders, inflammatory and pain conditions, and ophthalmic diseases [2]. The main categories of LAIs include aqueous suspensions or nanosuspensions, oil-based depot injections, polymer-based systems such as poly(lactide-co-glycolide) (PLGA) microspheres, and in situ forming implants (ISFIs), and pre-formed implants that may be biodegradable or non-biodegradable [3]. These platforms enable different types of release control, allowing the formulation approach to be adjusted according to the drug properties and therapeutic needs of the condition [4].

**Figure 1:**
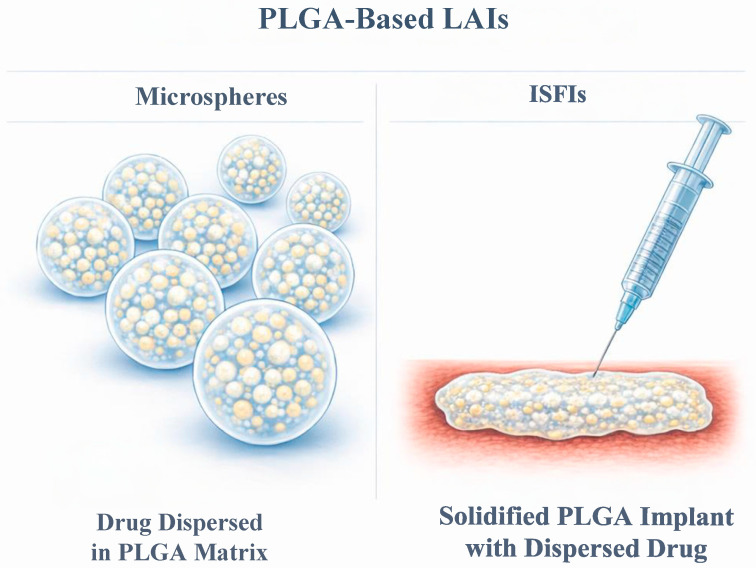
Major PLGA-based LAI delivery systems: microspheres and ISFIs (AI-assisted drawing using Microsoft Copilot, April 2026).

PLGA is the dominant polymer platform for LAI systems due to its biocompatibility, regulatory acceptance, and tunability through the hydrophilic (glycolic)/hydrophobic (lactic) monomer ratio, molecular weight, and end-group chemistry [5,6]. Two major PLGA-based LAI delivery systems are microspheres and in situ forming implants (ISFIs) (Figure 1).

Microspheres, which can be manufactured via different technologies, exhibit distinct internal porosity, morphology, and drug distribution, all of which directly influence encapsulation efficiency and release behavior [7]. ISFIs, by contrast, consist of PLGA dissolved in water-miscible organic solvents (e.g., N-methyl-2-pyrrolidone, dimethyl sulfoxide) that undergo solvent exchange and polymer precipitation after injection, forming depots whose solidification and release characteristics depend on polymer composition and solvent hydrophilicity [8]. PLGA biodegrades through hydrolytic cleavage of ester bonds followed by autocatalytic erosion, with faster degradation observed for lower-molecular-weight polymers, higher glycolide content, or more hydrophilic end groups [6]. The end products of PLGA degradation are lactic and glycolic acids, naturally occurring compounds that are eliminated through normal physiological pathways, including the tricarboxylic acid cycle and renal excretion [9].

Drug release from PLGA-based systems typically follows a triphasic profile comprising (i) an initial burst, (ii) a lag phase, and (iii) a secondary release phase. Furthermore, release from the PLGA LAIs can be controlled by at least three major mechanisms, or combinations thereof: diffusion through the polymer matrix, water-mediated transport processes, and polymer hydrolysis and erosion [10,11,12]. The balance among these mechanisms differs between microspheres and ISFIs. Microspheres, owing to their pre-formed microstructure, generally yield more predictable multiphasic profiles. In contrast, ISFIs often show a larger and more variable initial burst driven by rapid solvent efflux and crust formation, followed by diffusion- and erosion-controlled release due to the evolving depot morphology [8]. Overall, drug release from PLGA systems is inherently complex because it involves a multiphasic, dynamic process driven by the simultaneous interplay of numerous physicochemical and biological processes.

The in vitro release (IVR) test is a key test of drug product performance, and may serve several purposes. It is used as a tool for formulation development and process optimization, as well as for batch release, evaluation of drug product stability, life-cycle management, and the establishment of IVIVC [13,14,15]. Understanding the factors influencing drug release from both in vivo and in vitro perspectives is essential for the development of meaningful in vitro release tests and specifications [16]. Considering the complexity of drug release mechanisms for LAI products and the diversity of formulation design approaches, it is not possible to devise a universal one-size-fits-all IVR method applicable to all LAI products [4]. Currently, there are no standard compendial in vitro release methods for LAIs. Various IVR methods using both compendial and noncompendial setups have been explored for PLGA-based LAIs [4,17]. This article provides an overview of IVR methodologies for PLGA-based LAIs from academic, industrial, and regulatory perspectives to support future method development and standardization.

## 2. Drug Release Mechanisms from PLGA-Based LAIs

PLGA-based systems typically show a triphasic drug release profile comprising (i) an initial burst, (ii) a zero-order release phase, and (iii) a final rapid release phase. The relative contributions of the three phases may vary significantly, and some phases may be negligible in certain drug delivery systems [10,12].

The initial burst release from PLGA systems is related to the drug adsorbed onto the system’s surface and to the drug present in channels/pores that have direct access to the surface. Upon contact with the release medium, these drug molecules are rapidly dissolved and released. The burst release is generally higher for smaller microparticles than for larger ones (if their composition and structure remain the same), as the probability of the drug particle having direct access to the surface is higher in smaller microparticles. In the case of pre-formed PLGA implants, the surface-to-volume ratio and porosity are usually much lower compared to PLGA microparticles, and therefore, the burst release from these systems is often very limited. However, the burst release from in situ forming implants is usually much higher and more variable than that observed with pre-formed implants, as ISFIs are still liquid or semisolid at early time points (leading to greater drug mobility and potentially convective mass transport) [8,12].

The second phase (zero-order release) is currently the least well understood. In this phase, water wets the system, leading to PLGA ester bond cleavage throughout the system and bulk erosion. The surface of the system, which is in direct contact with the aqueous medium, may be transformed into a highly swollen PLGA gel (“corona”). The drug molecules located in the swollen PLGA layer may easily diffuse out of the system, and the swelling front likely moves at a very low, approximately constant rate. The swollen PLGA layer may also be seen as a “skin” (film or membrane) that functions as a diffusion-controlled barrier, resulting in zero-order drug release kinetics [5,12].

The final rapid release phase is initiated by substantial swelling of the entire PLGA system. Namely, due to the bulk erosion process, the PLGA system becomes more hydrophilic (ester-bond cleavage creates new hydrophilic end groups: -OH and -COOH) and less mechanically stable (the molecular weight of PLGA chains and the degree of chain entanglement decrease). Increasingly more water-soluble degradation products are formed, creating increased osmotic pressure and attracting water from the surrounding environment. Consequently, substantial amounts of water are driven into the system and the remaining undissolved drug particles can dissolve, leading to complete drug exhaust [12].

In the case of ISFIs, the drug release is typically biphasic, comprising (i) an initial burst, related to drug diffusion during implant formation, and (ii) a secondary slower release phase, where the drug substance is released from the solidified implant by a combination of diffusion and polymer erosion [18]. Although the dominant type of erosion for PLGA-based systems is bulk erosion, some PLGA systems may also exhibit certain features of surface eroding systems [12,19].

The drug release kinetics from PLGA systems are dependent on the type/grade of PLGA (molecular weight, lactic-to-glycolic monomer ratio, type of end groups, polymer architecture), physical state of the drug (dissolved or non-dissolved; amorphous or crystalline), physical state of the polymer, inner and outer structure of the dosage form (e.g., particle size, porosity) and the environmental conditions (e.g., pH and volume of medium, temperature, degree of agitation, presence of enzymes or inflammatory reaction). Furthermore, in the case of ISFIs, the surface area of the formed implant can have a significant impact on the solvent exchange kinetics and the associated burst release, as well as on the polymer degradation kinetics and the secondary release rate [12,18].

A multitude of processes may be included in the release kinetics of PLGA systems, including (but not limited to) wetting of the dosage form, desorption of the drug from the system’s surface, penetration of water into the system, drug dissolution, drug diffusion, ester hydrolysis, polymer swelling, pore closure, plasticizing effects, fusion of microparticles into lumps, osmotic effects, autocatalytic effects, saturation effects, drug-polymer interactions, etc. The relative importance of these phenomena may vary depending on the qualitative and quantitative composition of the drug delivery system, dimensions, geometry, inner structure, and manufacturing process. Often, only a few of these processes are dominant or release rate controlling for a specific PLGA-based delivery system [12].

Overall, drug release from PLGA systems is inherently complex and governed by formulation attributes, polymer properties, and physiological conditions at the administration site (in vivo) or release testing conditions (in vitro). An excellent overview of the current knowledge in the field of release mechanisms of PLGA-based drug delivery systems has recently been published by Siepmann and Siepmann [12].

## 3. Experimental Setups for In Vitro Release Testing of PLGA LAIs

One of the major challenges in the development of LAIs is the lack of compendial in vitro release testing methods [6]. As a general recommendation for IVR method development, existing compendial apparatuses should be used as a first approach, as this facilitates standardization and comparability between laboratories [16]. However, the standard pharmacopoeial apparatuses that are most commonly used for dissolution or in vitro release testing (i.e., basket and paddle apparatuses according to Ph. Eur. and USP) were originally developed for oral dosage forms and, in many cases, may not be appropriate for testing the parenteral dosage forms. Therefore, for some parenteral dosage forms, modified compendial or non-compendial apparatuses may be required [15].

USP <1001> *In vitro release test methods for parenteral drug preparations* provides guidance on methods that have been demonstrated as useful for IVR testing of parenteral products. It provides examples of apparatuses that have been applied to various parenteral products, including microparticles (Apparatuses 2 and 4, dialysis cell, incubation jar) and in situ forming preparations and implants (Apparatuses 2, 4, and 7, incubation jar) [15]. Further insight into the types of apparatuses and methods currently applied to LAIs can be obtained from the methods described in the USP Dissolution Methods Database (Table 1) and the FDA Dissolution Methods Database (Table 2) [20,21]. The recommendations from Ph. Eur. 5.17.1 *Recommendations on dissolution testing* and USP <1092> *The dissolution procedure: development and validation*, although originally developed for oral dosage forms, may also be useful when developing an in vitro release test procedure for parenteral dosage forms [15,22,23].

Importantly, in vitro release kinetics are highly dependent on the selected test method and experimental conditions. For example, Garner et al. investigated the impact of in vitro testing conditions, such as vessel dimensions, agitation speed, the presence of solid beads in the vessel, and media exchange volume, on the in vitro release rate from PLGA microparticles. Their results demonstrated that significantly different release profiles can be obtained for the same formulation simply by modifying IVR testing conditions. In fact, the effects of these methodological differences were so pronounced that they outweighed differences between formulations or process variants [27]. Furthermore, the Schwendeman research group has systematically investigated the impact of release media composition on the release rate from PLGA microparticles. The effects of pH, buffering species, and the presence of plasticizers in the release medium were evaluated. These studies demonstrated that the composition of the release medium can be manipulated to alter the drug release rate from PLGA microparticles, but also to change the underlying release mechanism(s) [11,28].

A variety of different in vitro release methods for PLGA-based LAIs have been described in the literature, using both compendial (pharmacopoeial) and non-compendial (non-pharmacopoeial) apparatuses. The most commonly used approaches can be grouped into three broad categories: sample and separate methods, dialysis methods, and flow-through methods. Each of these methods has its own advantages and limitations, as described in the following subsections and summarized in Table 3.

### 3.1. Sample and Separate Method

**Figure 2:**
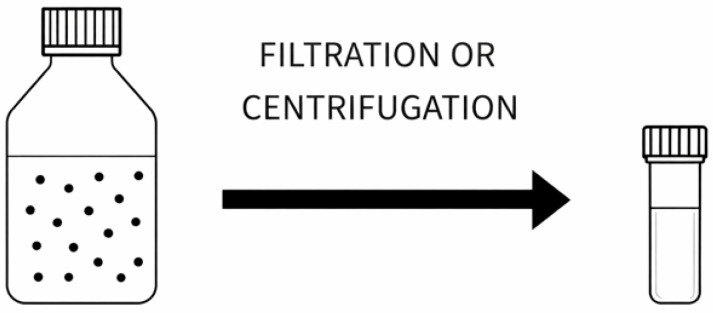
Schematic representation of the experimental setup for sample and separate method (AI-assisted drawing using OpenAI, February 2026).

Generally speaking, the sample and separate (SS) method is the simplest and most commonly used approach for dissolution or in vitro release testing. In this method, the sample is placed in a container filled with release medium, and drug release is assessed over time (Figure 2).

The types of containers vary widely between studies and may include vials, tubes, flasks, jars, or bottles [10,27,29,30]. The selection of the container is primarily guided by the required volume of release medium, which typically ranges from approximately 1 mL [31,32,33] to about 1000 mL [21]. It should be noted that the pharmacopoeial paddle and basket apparatuses also operate according to the sample and separate principle.

The release medium in the containers may be agitated using different techniques, including shaking water baths [34], orbital shakers [27,28,33], reciprocating shakers [35], magnetic stirrers [36], tube rotator systems [37], or paddle rotation in compendial paddle apparatuses [19]. Samples are withdrawn at predefined time points, followed by filtration or centrifugation to separate the release medium from undissolved material, and subsequent quantification of the released drug substance in the supernatant or filtrate. The selected filter should not adsorb the drug substance [23]. To maintain sink conditions, medium replacement is often performed after sampling.

Several PLGA-based systems with in vitro release methods described in the USP or the FDA Dissolution Methods Database are tested using the sample and separate approach. For example, the USP monograph for Goserelin implants describes an IVR test performed using flat-bottom glass jars, whereas FDA-recommended methods for Triamcinolone acetonide intra-articular suspension and Triptorelin pamoate intramuscular suspension employ a paddle apparatus [20,21].

The main advantages of the SS method include its simplicity, wide availability of the experimental setup, and high flexibility in release medium volume and equipment selection. Small containers and low media volumes are advantageous when low sample quantities are available or in cases of limited analytical sensitivity. Additionally, the use of small-volume tubular containers may facilitate better control of PLGA implant formation [38]. On the other hand, larger containers and higher medium volumes may be employed to ensure sink conditions for poorly soluble drug substances. Medium evaporation can be minimized by using well-closed medium containers. Overall, the SS technique is cost-friendly, easy to set up, and suitable for long-term studies [4,19,27,39,40].

However, there are also several disadvantages of the SS method, including potential floating of microparticles [34,41] and microparticle aggregation [39,42]. Aggregation of microparticles can be mitigated by the addition of surfactants [43,44] or by optimization of the agitation method [39,45]. Furthermore, the tendency for microparticle aggregation may be impacted by container geometry, as demonstrated by Garner et al. [27]. The experimental conditions should be optimized to eliminate aggregation, as it may lead to poor reproducibility, slower release rates, and incomplete release profiles [27,34].

When the SS method is applied to microparticle systems, the sampling technique can become cumbersome, with potential clogging of the filters [39] and unintended withdrawal of microspheres during sampling [42,46]. Microspheres removed from the system (e.g., adhering to filters or sampling probes) need to be reintroduced during medium replacement [34]. Sample loss or particle floating may further compromise reproducibility [34,41]. In addition, centrifugation-based separation requires subsequent resuspension of microparticles in the medium by shaking or vortexing, which may be difficult because of microparticle aggregation [36]. Finally, the wide variety of medium container dimensions and agitation methods limits inter-laboratory comparability of SS-based IVR data [17,27]. In order to standardize the experimental setup, the pharmacopoeial paddle apparatus (Apparatus 2) may be used in combination with standard vessels or mini vessels.

### 3.2. Flow-Through Method

**Figure 3:**
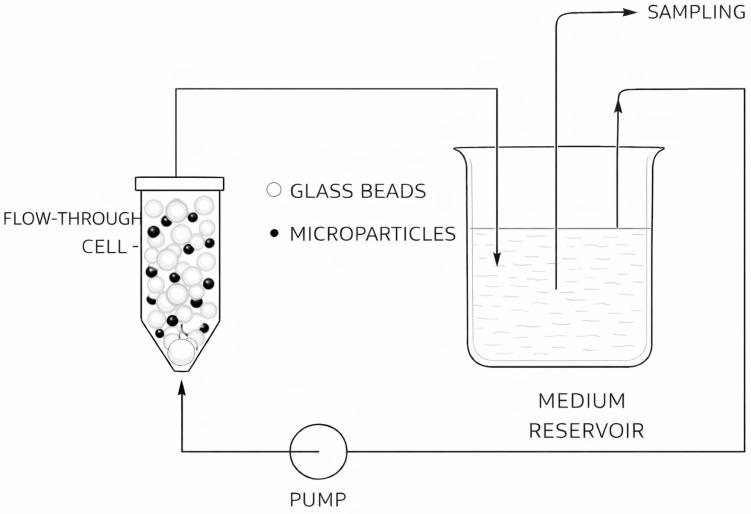
Schematic representation of the experimental setup for flow-through method (AI-assisted drawing using OpenAI, February 2026).

The setup for the flow-through method includes a medium reservoir, a pump, a flow-through cell, a filtering system positioned on top of the flow-through cell, and a sample collector (Figure 3).

The tested LAI product is introduced into the flow-through cell, and the medium is continuously pumped through the cell, followed by analysis of the eluent. The flow-through apparatus mimics the injection site with respect to immobilized microparticles and the limited volume of medium inside the flow-through cell, while the continuously circulating medium simulates the dynamic in vivo environment [17,47].

Specifications for flow-through cell size are described in pharmacopeias (Apparatus 4, according to Ph. Eur. and USP) [48,49]. Apparatus 4 can be operated in open- or closed-loop mode, with different cell sizes, flow rates, and temperatures. In the open-loop configuration, fresh medium continuously flows through the cell, and samples are collected using a fraction collector. In the closed-loop configuration, a fixed volume of medium is recirculated through the cell. In addition, the closed-loop setup minimizes medium evaporation during prolonged testing [50].

An example of the flow-through method using Apparatus 4 in closed-loop mode is described in the FDA Dissolution Methods Database for risperidone extended-release suspension [21].

Hydrodynamics within the flow-through cell may be modified by filling the cell with glass beads to achieve laminar flow or by removing them to allow turbulent flow. Microparticles are commonly layered between or mixed with glass beads to prevent aggregation and to achieve laminar flow within the cell [42,51,52]. For example, Rawat et al. described a sample preparation technique in which microspheres were divided into three approximately equal portions and layered between 1-mm glass beads in a 12-mm flow-through cell. An anti-static gun was used to neutralize static charge on glass beads, microspheres, and the spatula to facilitate sample preparation [53]. The ratio of microparticles to glass beads should not be excessive in order to avoid back-pressure issues [42].

On the other hand, Wang et al. investigated in vitro release from in situ forming risperidone and naproxen depots using flow-through cells operated with or without glass beads. The presence of glass beads caused mechanical compression and fragmentation of the PLGA matrix, resulting in accelerated degradation-driven release at later stages of the release profile while producing a lower initial release compared to bead-free conditions. Conversely, bead-free conditions preserved an intact, disk-like implant morphology and resulted in a more gradual release profile [54].

Compared with various non-compendial setups, IVR methods employing pharmacopoeial flow-through cells offer improved reproducibility, as they are based on a standardized apparatus with defined geometry and hydrodynamics, enabling meaningful inter-laboratory comparison [44]. Furthermore, the drug formulation is contained in the flow-through cell, making sampling and media replacement easy (i.e., there is no need for additional separation of the formulation from the medium containing the dissolved drug substance). Sampling convenience may be further enhanced using commercially available automated systems [39]. Medium volume can be readily adjusted to maintain sink conditions for poorly soluble drug substances or reduced when analytical sensitivity is limited [42,53]. Furthermore, flow-through cells may be coupled with in situ fiber-optic UV probes, since the dispersed microparticles are enclosed inside the flow-through cell and therefore do not interfere with UV analysis. This is particularly advantageous for the characterization of the burst release phase, as in situ measurement enables the easy collection of multiple frequent data points [42].

The main disadvantages of flow-through methods include the complexity of the apparatus [40] and the cumbersome setup procedure [55]. This method requires expensive and maintenance-intensive equipment that may not be readily available in many laboratories [27,40,56]. In addition, potential filter blockage may cause variations in flow rate and pressure buildup within the system [15,39,50]. Failure of O-rings or filters may occur during prolonged release testing [17]. Furthermore, adsorption of the analyte onto hydrophobic surfaces of the flow-through apparatus may occur; this effect can be mitigated by incorporating surfactants into the release medium [46].

Except for the compendial Apparatus 4, alternative non-compendial flow-through cell designs have been described in the literature, primarily to improve the biorelevance of IVR methods [57,58].

### 3.3. Dialysis Method

**Figure 4:**
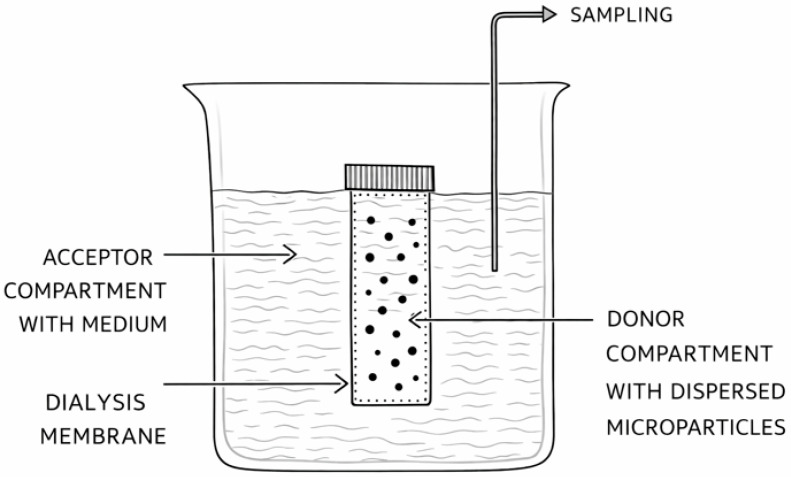
Schematic representation of the experimental setup for dialysis method (AI-assisted drawing using OpenAI, February 2026).

In the dialysis method, the test sample is introduced into a dialysis bag (donor compartment), sealed, and placed in a vessel containing the release medium (acceptor compartment) (Figure 4).

The dialysis bag is composed of a semipermeable membrane that allows diffusion of the drug substance from the donor compartment into the acceptor compartment. Drug release is monitored by sampling from the acceptor compartment.

The membrane material and pore size must be carefully selected. The drug substance should not bind to the selected membrane [39]. The pore size of the dialysis membrane is characterized by its molecular weight cut-off (MWCO). Dialysis membranes with sufficiently high MWCO values are used for in vitro release studies to ensure that they are not a limiting factor for drug diffusion [39,56]. It has been reported that the MWCO should be approximately 100-fold higher than the molecular weight of the investigated drug substance [59]. Dialysis membranes should be pretreated according to the manufacturer’s instructions prior to use.

The volume of the release medium in the acceptor compartment should be at least 6–10 times greater than the volume inside the dialysis bag to maintain a sufficient concentration gradient and to provide a driving force for drug transport across the membrane [39]. Agitation of the outer medium is commonly applied to minimize the unstirred-water-layer effect. Various modes of agitation may be used, including a magnetic stirrer, shaker, or compendial paddle apparatus [36,39].

Several commercially available dialysis devices have been employed for in vitro release testing of PLGA-based systems, including Float-A-Lyzer [60], Slide-A-Lyzer MINI [61], Tube-O-DIALYZER [62], and the Dispersion Releaser [63]. Float-A-Lyzer (Repligen, USA) is a ready-to-use dialysis device equipped with a flotation ring and cellulose ester membrane, available in a wide range of MWCO values (0.1–1000 kDa) and volumes (1–10 mL). These devices may be used in combination with pharmacopoeial Apparatus 1 or 2. Slide-A-Lyzer MINI (Thermo Fisher Scientific, USA) is a disposable polypropylene cup (0.1–2 mL) with a regenerated cellulose membrane of various MWCOs (2–20 kDa) and can be placed in conical or microcentrifuge tubes or alternatively used with a floating unit and a larger beaker. Tube-O-DIALYZER (G-BioSciences, USA) utilizes a regenerated cellulose membrane with MWCOs ranging from 1 to 50 kDa and accommodates sample volumes of 20 μL to 2.5 mL.

**Figure 5:**
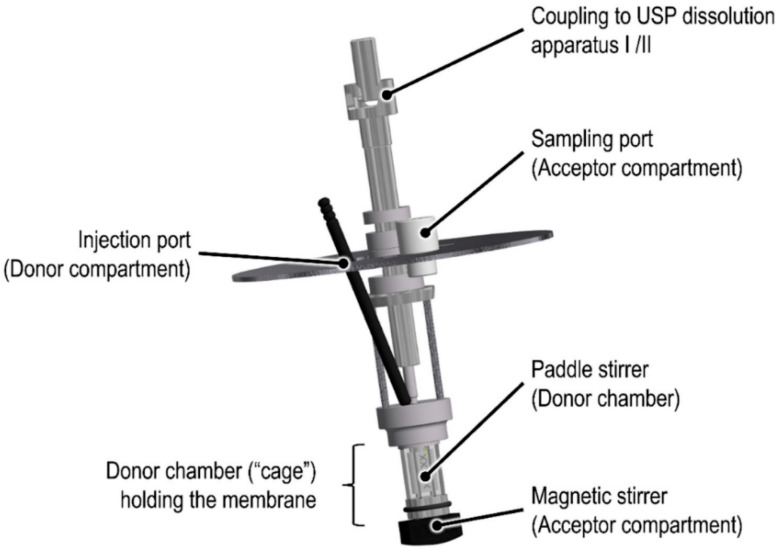
Schematic of the commercial Dispersion Releaser PT-DR (by Pharma Test Apparatenbau AG, Germany). Reprinted from [65] “An Update to Dialysis-Based Drug Release Testing—Data Analysis and Validation Using the Pharma Test Dispersion Releaser” by M.-P. Mast, H. Modh, J. Knoll, E. Fecioru, M.G. Wacker, 2021, Pharmaceutics, 13 (12), 2007, https://doi.org/10.3390/pharmaceutics13122007. CC BY 4.0.

The Dispersion Releaser (Pharma Test, Germany) (Figure 5) is a dialysis-based device designed for use with pharmacopoeial Apparatus 2 and standard or mini dissolution vessels. In this setup, the dissolution vessel represents the acceptor compartment, while the donor compartment consists of a dialysis membrane mounted around a cylindrical housing and sealed with two O-rings. The donor compartment is continuously agitated by a paddle stirrer equipped with a permanent magnet, and the propulsion is transmitted to a magnetic stirring device in the acceptor compartment [40,63,64].

It is important to note that there are two kinetics involved in the total drug release rate determined by dialysis methods, namely the rate of drug substance release from the carrier and the permeation rate through the dialysis membrane. Consequently, the dialysis membrane may limit the sensitivity of the method due to a delay in membrane transport [40,66]. For this reason, it is recommended to conduct membrane permeability experiments during membrane selection, in order to yield an optimized dialysis method that enables rapid transport of the released drug into the acceptor compartment [40,65]. Membrane permeation rate is determined in a reference experiment performed with the drug substance solution. Subsequently, this membrane permeation rate may be used for normalization of release profiles through mathematical modeling approaches, such as the four-step model [43,64].

Compared with sample and separate methods, dialysis techniques do not require physical separation of the released drug substance from microparticles. Sampling and medium replacement are simplified due to the physical separation of microparticles by the dialysis membrane [39]. In addition, dialysis setups more closely simulate in vivo conditions following subcutaneous or intramuscular administration, where microspheres are immobilized within the tissue rather than freely dispersed in the medium [39,50]. In the case of in situ forming implants, dialysis methods may lead to standardized implant geometries and reduced variability in release profiles [67].

The disadvantages of the dialysis method include a cumbersome setup procedure for dialysis bags and the use of non-standardized apparatuses, which makes comparison between laboratories difficult. These issues may be overcome by using a commercially available dialyzer, which in turn simplifies and standardizes the setup and facilitates inter-laboratory comparison [39,55,61,65]. In addition, the lack of agitation in the donor compartment may lead to microparticle aggregation and poor reproducibility of the release data [41,68]. This issue has been addressed in the commercially available Dispersion Releaser device, where continuous agitation of the donor compartment is applied [63,64].

The small volume of medium within the dialysis bag and the limited membrane surface area may potentially lead to a violation of sink conditions in the inner compartment if the drug release from the carrier is faster than drug diffusion through the membrane [17,67]. To address this problem, a reverse dialysis configuration has been proposed, in which the dialysis bag serves as the acceptor compartment while microparticles are suspended in the outer medium [17,69].

Furthermore, equilibration between the donor and acceptor compartments is slow in setups with a small membrane surface area (e.g., membrane at one end of the tube), which may hinder accurate characterization of the initial burst effect. This issue may be addressed by using a dialysis setup with a large surface area to facilitate drug transport [36,39].

Finally, the dialysis method is not suitable for drug substances that bind to the dialysis membrane [39].

### 3.4. Methods for Implant Formation Within IVR Testing of In Situ Forming Implants (ISFIs)

PLGA-based ISFIs are typically composed of PLGA polymer dissolved in an organic solvent (e.g., NMP, DMSO, triacetin) and the drug substance, which may be suspended or dissolved in the drug product. Upon injection at the administration site, these systems form a drug-releasing implant via phase inversion. In this process, the sol-to-gel transition is obtained by solvent exchange, whereby the organic solvent diffuses out of the polymeric system, while aqueous medium enters the system, causing water-insoluble polymers to precipitate and entrap drug substances within the formed implant.

Importantly, unlike the evaluation of pre-formed implants, ISFI testing involves in situ formation of the implant within the IVR test. The in vitro formation of the implant, including factors such as injection parameters, resulting depot geometry, surface-to-volume ratio (S/V), and initial water uptake, critically determines its microstructure and consequently has a significant impact on the release profile. Several recent FDA-funded studies have emphasized that controlling depot formation during IVRT is the key determinant for achieving reproducible, discriminating, and potentially biopredictive IVR methods [8,67,70,71,72].

An early study recognized the importance of uniform implant preparation and tested IVR of thin-layer implants prepared using a homemade holding cell. This manner of implant preparation was sufficient to discriminate among PLGA ISFIs containing leuprolide acetate prepared with different molecular weights [73]. In this setup, a mesh separated the holding cell from the release medium, allowing diffusion and phase inversion processes to occur while preserving the physical integrity of the forming implants and resulting in low IVR variability [73].

Zhang and Fassihi systematically investigated the impact of the implant formation method on the in vitro release rate of the PLGA-based ISFIs containing naltrexone [62]. Three different formation methods were used, and the formed implants were tested using the sample and separate method. Conventional injection of PLGA/NMP formulations into aqueous media resulted in unpredictable and non-uniform implant shapes due to rapid phase inversion. Injection through a needle produced thin, fragile, helix-like filaments with highly variable S/Vs, which resulted in poor reproducibility. Injections without a needle yielded irregular spheres prone to entrapping air bubbles. These voids altered local density, caused flotation, and generated microenvironmental differences that accelerated or disrupted diffusion. Both of these methods of implant formation led to considerable variability in early burst release, overall release kinetics, and long-term depot integrity. To overcome geometry-related variability, the authors introduced a mold-based method for creating disk-shaped implants (1.4 cm-diameter cylindrical cavity with a 1.5 cm depth). The key characteristics of this procedure included defined dimensions of the depot, controlled injection force to simulate physiological resistance during subcutaneous injection without distorting the implant, and immediate solidification since the polymer solution formed a uniform “gel disk” upon contact with aqueous buffer. This controlled depot preparation eliminated air entrapment, standardized the S/V, and resulted in reproducible release profiles across replicates. This study showed that standardizing depot geometry is a prerequisite for increasing reproducibility of the IVR method [62].

In situ forming implants prepared using different formation methods, resulting in significantly different implant geometries, internal microstructures, and drug release kinetics, were tested by Suh et al. [67]. Using leuprolide acetate in PLGA/NMP systems, the study evaluated five depot formation approaches, with in vitro release testing performed using the sample and separate method. The authors noted that smaller injection volumes (approximately 50 µL) are more manageable when administered directly into the IVR medium, as they tend to form relatively uniform depots. In contrast, larger volumes of ISFIs, as tested in this study (250 µL), result in greater variability due to increased implant size. Therefore, the depot preparation step was found to be more critical for achieving consistently shaped implants in the case of higher ISFI dosing volumes [67].

In vitro formation methods resulting in irregular implant geometries (medium injection, flash freezing, gelatin capsule) substantially elevated the initial burst release due to uncontrolled expansion or structural breakage. In contrast, controlled-geometry methods (dialysis sacs and water-soluble PVA thin film sacs) maintained a reproducible implant size and surface area, resulting in significantly reduced, more consistent burst release. Furthermore, microstructural imaging confirmed that controlled-geometry methods generated more uniform solid/semi-solid phase distributions, while irregular-geometry methods produced heterogeneous architectures related to variable release [67].

In a notable study, Wang et al. developed a new adapter-based method using water-soluble polyvinyl alcohol (PVA) films combined with a Teflon frame as a PVA carrier to improve in vitro release testing for in situ forming implants. Upon contact with an aqueous medium, uniform depots are formed, whereby the PVA film dissolves. To evaluate the proposed adapter, two widely different implant types were selected: a risperidone formulation, in which the drug remains suspended in the PLGA/NMP matrix, and a naproxen formulation, in which the drug fully dissolves in the same polymer/solvent system [54].

For risperidone formulations, the adapter-based method formed uniform, disk-like depots that resembled the implants obtained in the in vivo testing in rabbits, improving the variability observed with direct injection. Adapter preparation captured three distinct release phases for risperidone implants: burst, diffusion-controlled release, and degradation-controlled release. In contrast, direct injections produced irregular implant geometry and resulted in apparent zero-order release. The IVR method with the proposed adapter showed discriminatory ability, differing between risperidone formulations with different PLGA ratios and molecular weights. Release profiles were reproducible across different sample injection volumes (0.1–0.6 mL), which was attributed to a constant diffusional path length and surface area per unit mass [54].

In the same study, naproxen formulations were prepared as solutions in PLGA/NMP systems. Upon formation within the adapter, naproxen depots exhibited consistent shape and minimized variability compared with direct injection. Naproxen release followed first-order kinetics regardless of the applied IVR method, indicating diffusion-dominated behavior with rapid solvent exchange. Release profiles were reproducible across different sample injection volumes (0.1–0.6 mL). The adapter reduced variability in both drug and NMP release profiles relative to the direct injection method. Differences between naproxen formulations with different PLGA grades were also observed, confirming adequate discriminatory power. Reproducibility was satisfactory across repeated studies and different adapter volumes, confirming the robustness of the method for solution-type ISFIs [54].

A follow-up study from the same group compared different implant formation methods applied to ISFIs with distinct drug release mechanisms, namely PLGA/NMP suspensions containing meloxicam or risperidone and a PLGA/NMP solution containing naproxen [19]. The study evaluated three depot formation methods: direct injection, the PVA film combined with a Teflon adapter, and the GF/F glass fiber membrane combined with a Teflon adapter. The PVA–Teflon adapters performed well in combination with naproxen and risperidone depots, which follow bulk-erosion-driven release and remain structurally intact during the IVR test, maintaining consistent depot geometry and producing low-variability release profiles. In contrast, meloxicam depots underwent surface erosion and progressively thinned and fragmented when formed with the PVA–Teflon adapter, causing variability due to uncontrolled detachment of fragments. Using the GF/F–Teflon adapter prevented fragmentation and stabilized depot geometry, resulting in more reproducible meloxicam release despite late-stage GF/F membrane degradation [19].

A complementary study from the same group systematically examined how in vitro implant formation conditions affect risperidone release from PLGA/NMP ISFIs and aimed to establish an IVIVC with a rabbit subcutaneous model [71]. Three methods of implant formation in the IVR test were developed to independently modulate depot morphology, exposed surface area, mechanical confinement, water uptake, and phase inversion kinetics. These methods used a PVA-film/Teflon adapter, a GF/F-membrane/Teflon adapter (sealed and half-open), and a sandblasted glass slide ring as molds for controlled implant formation.

The PVA-film method enabled unrestricted swelling and generated reproducible but non-biopredictive release characterized by a minimal burst, a prolonged lag phase, and accelerated degradation-driven release. Introducing GF/F glass fiber membranes allowed controlled confinement and tunable surface-to-volume ratios through sealed and half-open formats; however, membrane integrity was occasionally compromised at high loading, and mechanical pressure introduced artificial constraints not reflective of physiological conditions. In contrast, the sandblasted glass-slide ring method provided precise shape control without imposing mechanical stress, maintained depot adhesion during agitation, and ensured consistent geometry with high water accessibility. This setup enabled accurate adjustment of surface-to-volume ratios (20–60 cm^−1^) and avoided confounding effects from swelling limitations or structural failure. In vivo, the risperidone implant formed a thin disk-shaped depot with a high surface-to-volume ratio (~50–60 cm^−1^), rapidly released approximately 90% of NMP within 24 h, and exhibited a biphasic profile driven initially by diffusion and later by polymer degradation. Among the tested systems, the glass-slide ring method most closely reproduced these physiological conditions by matching the in vivo surface-to-volume ratio and permitting unhindered water uptake and phase inversion, thereby providing the best in vitro–in vivo alignment [71].

In the study by Karp et al., in vitro implants were produced using a custom flat holding-cell mold that generated uniform, thin, disk-shaped PLGA–progesterone implants [74]. The authors investigated release mechanisms, including diffusion, dissolution, and swelling, across formulations differing in solvents, PLGA concentration, and drug load. A defined amount of formulation was applied into the mold, and controlled exposure to a buffer triggered phase inversion, yielding reproducible implants with consistent geometry. Phase inversion occurred over 20 h, after which the implants were considered fully formed. Because of this, the initial burst that occurred during the implant formation period was not captured in the in vitro release test. Instead, early drug release was captured indirectly through drug entrapment measurements. This standardized setup minimized variability and allowed clear discrimination among formulations in the post-burst phase [74].

**Figure 6:**
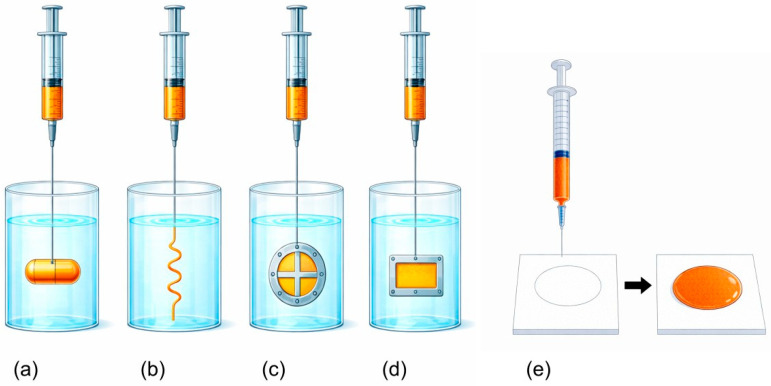
Implant formation methods for ISFIs: (**a**) injection into the empty capsule, (**b**) free injection, (**c**) injection into Teflon adapter with PVA film, drawn based on [54], (**d**) injection into dialysis cassette, (**e**) injection into sandblasted glass slide, drawn based on [71] (AI-assisted drawing using Microsoft Copilot, April 2026).

Overall, these studies demonstrate that the method of implant formation is a primary determinant of IVR performance for ISFIs. Some of the approaches evaluated in this chapter are presented in Figure 6.

The choice of the formation adapter, the degree of mechanical confinement, and the ability to control the surface-to-volume ratio all play central roles in achieving reproducible release profiles, as summarized in Table 4. Importantly, adapting the depot formation method to match the physical characteristics of the depot formed in vivo may help mimic in vivo release and guide early formulation development.

### 3.5. Release Medium

Selection of the release medium is generally governed by drug solubility and stability over the duration of the IVR study [39]. For PLGA-based LAIs, the most commonly used medium for in vitro release testing is pH 7.4 phosphate-buffered saline (PBS), corresponding to the physiological pH at subcutaneous and intramuscular administration sites. In addition, pH 7.4 HEPES-buffered saline (HBS) has been reported as an alternative buffer system [11,28]. In some studies, water has been used as the release medium [33,76]. However, as water quality and pH may vary, its use is recommended only when it has been demonstrated that pH fluctuations do not influence in vitro release characteristics [22,23]. In addition, buffers with acidic or alkaline pH may be used as release media for accelerated IVR testing.

To ensure complete drug release for poorly soluble drug substances, release media may require supplementation with solubilizing agents, including surfactants or organic solvents [21,77]. Furthermore, when microparticles exhibit poor wettability, a small amount of surfactant may be added to the medium to minimize microparticle aggregation or floating [15,44]. The nonionic surfactant polysorbate (Tween 20 or Tween 80) is frequently used for IVR testing of PLGA-based microparticles, typically at concentrations ranging from 0.02% to 0.05% [10,29,30,44]. Beyond simple buffer systems such as PBS and HBS, more complex biorelevant release media are being developed in an attempt to simulate physiological conditions at the administration site.

Considering the usual duration of a long-term IVR test for LAI products (often weeks to months), special attention must be given to potential medium evaporation, microbial growth, and chemical degradation of the released drug substance in the medium [15]. Medium evaporation may be prevented by using sealed containers. When a poorly sealed apparatus is employed (e.g., pharmacopoeial paddle apparatus), an internal standard may be used to correct for changes in medium volume due to evaporation. In any case, it should be considered whether the decreased medium volume will affect sink conditions [15].

Microbial growth can be limited by adding preservatives to the release medium, such as sodium azide. Concentrations ranging from 0.01% to 0.15% sodium azide have been reported in the literature [15,19,27,29,78,79].

In cases where chemical degradation of the drug substance occurs in the release medium, the use of an alternative pH (at which the drug substance is more stable) or the addition of antioxidants (e.g., sodium ascorbate) may be beneficial. Complete medium replacement or use of a flow-through apparatus in an open-loop configuration may also be helpful, as the dissolved drug does not remain in the system for the entire duration of the test [15,34]. Drug degradation in the release medium may further be addressed by nonspecific analytical techniques, such as UV–Vis spectroscopy or HPLC with class separation, or by summation of peaks corresponding to the drug substance and its degradation products [15,58,80]. Alternatively, residual drug content in the microspheres may be quantified when instability in the release medium precludes direct measurement; however, this destructive approach requires a substantial amount of sample and is therefore not generally preferred [29,39,80].

IVR testing for quality control purposes is generally performed under sink conditions, meaning that the material already in solution does not exert a significant modifying effect on the dissolution rate of the remainder. Sink conditions normally occur when the volume of release medium is at least three- to ten-fold greater than the saturation volume [22]. Under sink conditions, IVR results are more likely to reflect the properties of the dosage form [23]. Although the volume of fluid at subcutaneous and intramuscular administration sites is small, there is constant fluid movement and replacement, as well as drug diffusion away from the site [44]. In other words, the tissues surrounding the injected drug delivery system may function as a sink [81]. Nevertheless, when conducting biorelevant release testing and simulating the physiological environment, the sink conditions cannot be assumed in vivo. Estimation of drug distribution between the administration site and systemic circulation should therefore be performed on a product-specific basis, and for some products, IVR testing under non-sink conditions may be warranted [40,50].

### 3.6. Temperature

In vitro release testing for LAIs intended for intramuscular or subcutaneous administration is generally performed at 37 °C. Elevated temperatures may be used for accelerated IVR testing. According to pharmacopoeial requirements, the permissible temperature range for in vitro release testing is ±0.5 °C [48,49]. However, for certain PLGA-based systems, even tighter temperature control may be needed during IVR testing. For example, Rawat et al. demonstrated that temperature variations of ±0.5 °C resulted in significant differences in release profiles of commercial risperidone PLGA microspheres, and this was attributed to temperature-dependent changes in risperidone-catalyzed PLGA degradation [53].

### 3.7. Accelerated Methods

LAIs are typically designed to deliver the drug substance over periods of weeks or months. Consequently, real-time in vitro release testing for these products also requires extended periods of time. Accelerated or short-term IVR testing is therefore considered a practical alternative, as it enables completion of release studies within days rather than months.

Development of an appropriate accelerated IVR method should be based on an understanding of the in vitro drug release mechanism. In addition, the impact of the applied accelerating conditions on the release mechanism should also be understood [47,50]. For example, drug release may be accelerated by the addition of organic solvents to the release medium, which can solubilize the PLGA polymer and shift the release mechanism from erosion/diffusion-controlled kinetics to diffusion-controlled kinetics. Furthermore, for PLGA-based systems exhibiting multiphasic release profiles (e.g., burst release, lag phase, and secondary release), some of the release phases may be diminished or lost entirely under accelerated conditions [51].

Dosage form attributes that should be considered during accelerated method development include matrix glass transition temperature, solubility of formulation components in the release medium, polymer degradation rate, and stability of the dosage form under accelerated conditions [50].

Accelerated IVR methods represent an attractive tool for rapid assessment of formulation and process changes during drug product development, as well as for batch release and quality control purposes [39,55]. However, a relationship between the real-time and accelerated release methods should be established in order to demonstrate the suitability of the accelerated method as a routine quality control test. Optimally, a 1:1 correlation is achieved between the real-time and accelerated release profiles [47]. The scaling factor between the real-time and accelerated release profile may be determined as the ratio of the time required to reach 50% drug release under real-time and accelerated conditions [53].

Ideally, an accelerated IVR test should increase the drug release rate without altering the underlying release mechanism. However, a change in release mechanism may be acceptable if the accelerated method demonstrates adequate discriminatory power and preserves the rank-order relationship between formulations when compared with the real-time method [4,53].

Accelerated release is typically achieved by modifying one or more experimental conditions applied in real-time IVR testing, including temperature, pH, agitation, and the addition of surfactants or organic solvents [47].

Elevation of temperature is the most frequently applied strategy to accelerate drug release from PLGA-based systems (Table 5). At elevated temperatures, polymer chain mobility increases, and this leads to accelerated drug release via diffusion. In addition, elevated temperature also increases polymer hydration and polymer degradation rate, thereby accelerating drug release via erosion [47,82]. However, it is generally recommended that the applied temperature should not exceed the glass transition temperature (Tg), as temperatures above Tg may alter the release mechanism [17].

In a study by Zolnik et al., accelerated testing at elevated temperatures has demonstrated a linear correlation with real-time data for erosion-controlled PLGA systems containing dexamethasone. On the other hand, the same accelerated release test appeared not to be suitable for formulations with dominantly diffusion-controlled release. Namely, the increased polymer mobility at elevated temperatures may lead to morphological changes, such as surface pore closure and microparticle aggregation, which in turn may hinder drug diffusion and decrease the drug release rate. Moreover, the applied accelerated test did not give an accurate prediction of the burst release phase, which is also diffusion-controlled. It was therefore recommended that the accelerated test should be supplemented by a real-time study to adequately assess the burst release phase [83].

Andhariya et al. developed an accelerated IVR test at 45 °C for naltrexone-loaded PLGA microparticles. The testing duration was reduced from 35–40 days (real-time test) to 6 days (accelerated test). A linear correlation between real-time and accelerated release profiles was observed, and the method was shown to be discriminatory toward manufacturing differences, which led to different particle size and porosity of the studied microparticle formulations [34].

Li et al. investigated accelerated IVR methods for PLGA-based ISFIs containing leuprolide acetate formulated with NMP, DMSO, or triacetin as cosolvents. Acceleration was achieved by increasing the test temperature to 50, 55, and 60 °C, resulting in a substantial shortening of the release duration from approximately 50 days at 37 °C to 7–14 days at elevated temperatures. An Arrhenius relationship between real-time and accelerated release data was established for NMP- and DMSO-based formulations, enabling prediction of zero-order release at 37 °C. Among the evaluated temperatures, 50 °C provided the best discriminatory ability, whereas higher temperatures diminished differentiation between formulations. In contrast, accelerated testing at elevated temperatures was not suitable for triacetin-based formulations, since triacetin hydrolysis to acetic acid caused a pronounced pH reduction, accelerating the PLGA degradation, altering the release mechanism, and preventing accurate real-time correlation [86].

In a study by Wang et al., increasing the temperature to 40 °C shortened the release duration of risperidone from PLGA/NMP ISFIs from approximately 45 to 25 days while preserving the real-time release mechanism, as demonstrated by the linear time-scaled correlation between the 37 °C and 40 °C profiles. However, the addition of 0.1% Triton X-100 at 40 °C accelerated water uptake and altered PLGA phase separation, which changed the relative contributions of diffusion and degradation to the overall release kinetics. As a result, the kinetics no longer matched real-time release, and no linear IVR correlation could be established [54].

Tipnis et al. explored the impact of temperature on the in vitro release of triamcinolone acetonide from PLGA microparticles. Surprisingly, it was found that drug release was slower at 39 °C than at 35 °C and 37 °C, and this was attributed to polymer plasticization at 39 °C, which in turn impacted the microparticle morphology. It was concluded that elevated temperature may not be a suitable parameter to accelerate drug release for all PLGA-based microparticle systems [79].

Modifications of the release medium composition, including changes in pH or the addition of organic solvents, have also been employed to accelerate drug release from PLGA-based systems. A change in medium pH can accelerate the hydrolytic degradation of PLGA. Both acidic and alkaline conditions catalyze ester hydrolysis and polymer degradation, and consequently, may accelerate drug release. However, compared with temperature elevation, pH changes typically result in only moderate acceleration of the release rate [47,51]. Importantly, polymer erosion mechanisms differ under acidic and alkaline conditions: acidic media promote bulk erosion similar to that observed at pH 7.4, whereas strongly alkaline conditions (pH > 13) induce surface erosion [87].

Organic solvents such as ethanol or acetonitrile have been used to accelerate drug release from PLGA-based systems [84,85,88]. Kamberi et al. demonstrated that acetonitrile increases the porosity of PLGA stent coating, leading to accelerated release [88]. Xie et al. developed an accelerated IVR method for thymopentin-loaded PLGA microparticles using a release medium containing 20% ethanol in combination with a gradient heating program. The temperature of the release medium increased in three steps, starting with 2 h at 40 °C, followed by 1 h at 45 °C, and finally 27 h at 50 °C. The testing duration was reduced from 30 days (real-time testing) to 30 h (accelerated testing). The developed accelerated method correlated well with the real-time release at 37 °C. In addition, it was found that the accelerated method with the gradient heating program (starting with a temperature of 40 °C) simulated well the burst release phase of the real-time test and that this approach may, in some cases, eliminate the need for an additional real-time study to assess the burst phase. Furthermore, the applied accelerated method was able to discriminate between formulations with different PLGA molecular weights and lactide-to-glycolide molar ratios [85].

Finally, it should be emphasized that elevated temperatures and extreme pH conditions may induce degradation of the drug substance [34,47,82]. Furthermore, the stability of the release medium components, as well as the robustness of the test apparatus under the applied accelerated conditions, should also be considered [47].

## 4. Method Validation and Discriminatory Power

In order to be used as a routine quality control test, the in vitro release method should be validated in accordance with the ICH Q2(R2) guidelines [89].

Rawat et al. described the validation of an accelerated IVR method for a commercial PLGA microsphere product (Risperdal Consta) using a flow-through apparatus [53]. The method was validated for robustness (with respect to small variations in method parameters) and reproducibility (with respect to different analysts and apparatuses). The authors demonstrated the robustness of the method to variations in flow rate, size of the flow-through cell, size of the glass beads, cell preparation technique, and the amount of microspheres loaded into the cell. In contrast, tight control of the temperature and pH of the release medium was identified as critical for achieving reproducible IVR profiles from the tested drug product. In addition, the authors highlighted the importance of medium deaeration at the beginning of the IVR test, as well as throughout the test, as re-aeration of the medium may occur over time and affect the release results [53]. It should be noted that complete validation of an IVR method for regulatory purposes would also need to cover validation of the analytical quantification step, including specificity/selectivity, accuracy, reportable range, and precision, in accordance with ICH Q2(R2) [89].

Furthermore, the discriminatory power of an IVR method intended for quality control purposes must be demonstrated. Discriminatory power refers to the ability of the test procedure to differentiate between batches manufactured with different critical process parameters and/or critical material attributes and/or critical process parameters, which may have an impact on bioavailability. In other words, the IVR test conditions should be chosen so that they, in combination with appropriately defined IVR specification limits, allow discrimination between acceptable and non-acceptable batches [15,23,90,91]. Additional guidance related to method validation and assessment of discriminatory power is available in the ICH Q2(R2)/ICH Q14 training materials [92].

The Burgess group has evaluated the discriminatory power of the IVR methods developed for PLGA microspheres containing various drug substances, including risperidone (poorly soluble small molecule), naltrexone (highly soluble small molecule), and leuprolide acetate (peptide). In these studies, IVR testing of risperidone- and naltrexone-loaded microspheres was performed using flow-through cells, whereas leuprolide-loaded microspheres were tested using a sample and separate method. In all cases, the IVR methods successfully discriminated between compositionally equivalent microspheres manufactured with deliberate process variations. Moreover, the in vivo pharmacokinetic profiles of the same microsphere formulations were determined following intramuscular administration in rabbits. In each of these studies, the in vivo release profiles of the process variants in the investigated animal model correlated well with the in vitro release profiles, and the authors reported that a Level A IVIVC was established [31,44,93].

Zolnik and Burgess further evaluated the discriminatory power of the IVR test method towards dexamethasone-loaded PLGA microspheres prepared with different polymer molecular weights. The proposed in vitro method was able to differentiate between the two tested PLGA microsphere formulations exhibiting distinct in vivo release characteristics [83].

Furthermore, the potential of an in vitro release method to be used as a stability-indicating test has been described in a study using risperidone-loaded PLGA microspheres, which were exposed to various storage conditions. After exposure of the microspheres to accelerated storage conditions, significantly faster in vitro release and a marked decrease in PLGA molecular weight were observed, confirming the discriminatory power of the applied IVR method towards potential stability-related changes for the tested drug delivery system [52].

## 5. Biorelevant Considerations Related to the Administration Site

**Figure 7:**
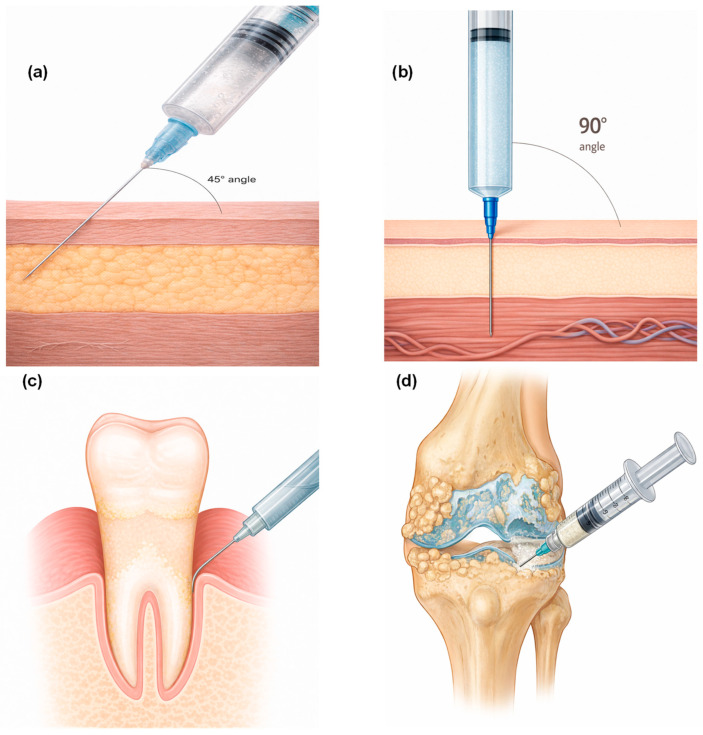
Schematic representation of the main administration routes for PLGA-based LAIs: (**a**) subcutaneous, (**b**) intramuscular, (**c**) subgingival, (**d**) intraarticular administration. (AI-assisted drawing using Microsoft Copilot, April 2026).

Subcutaneous (SC), intramuscular (IM), subgingival, and intra-articular sites (Figure 7) are the primary routes explored for administering PLGA-based systems, with SC and IM being the most widely utilized. Biorelevant IVR methods for LAIs aim to better reflect the physiological conditions at the site of administration. Incorporating site-specific characteristics into IVR method design enhances the approximation of in vivo drug release profiles and potentially enables more effective formulation screening in early development.

### 5.1. Subcutaneous (SC) Administration

The SC route of administration is characterized by the presence of adipocytes, an extracellular matrix (ECM) containing glycosaminoglycans such as hyaluronic acid (HA), a limited number of blood capillaries, and interstitial fluid. Due to reduced vascularization and a denser ECM, drug absorption in subcutaneous tissue typically occurs at a slower rate compared with IM administration. Interstitial fluid (ISF) in SC tissue is the first fluid to interact with injected drugs [94]. ISF is constantly exchanged with plasma and lymph, driven by hydrostatic and osmotic pressures. Lymphatic drainage is crucial for the clearance of large molecules and particles, and local tissue movement (e.g., muscle contractions, injection trauma) further influences fluid dynamics [77,95]. Moreover, proteases in the SC tissue can degrade peptides and proteins, affecting their bioavailability [77]. ISF composition is similar to that of plasma but with lower protein and lipid content due to the selective permeability of capillary walls [94,96]. Major electrolytes include Na^+^ (132–146 mM), K^+^ (10–21 mM), Ca^2+^ (0.7–3.2 mM), Mg^2+^ (0.7–1.8 mM), Cl^−^ (104–158 mM), and HCO_3_^−^ (20–28 mM). Protein levels range from 22 to 57 g/L, with albumin at 14–35 g/L, making up about 60% of total protein. Phosphatidylcholine and sphingomyelin are the main lipids in the ISF [94]. Gao et al. developed a Simulated Subcutaneous Interstitial Fluid (SSIF), a biorelevant medium that reflects the subcutaneous tissue by including major ions, buffers, and proteins. SSIF consists of sodium (136.0 mM), potassium (3.9 mM), calcium (1.3 mM), magnesium (0.5 mM), chloride (114.9 mM), bicarbonate (20.6 mM), phosphate (21.0 mM), sulfate (20.5 mM), acetate (5.0 mM), and Tris buffer (50.0 mM) and is further supplemented with 55% *v*/*v* fetal bovine serum to achieve a final protein concentration of 25 g/L [97].

In addition to using media compositions that replicate the characteristics of subcutaneous ISF, agarose gels have been used as media for IVR testing to simulate the architecture of subcutaneous tissue in vitro [86,98,99]. These agarose-based systems act as diffusion-dominated barriers, minimizing convective transport and more closely simulating the gel-like extracellular matrix of SC tissue [99]. Typical agarose concentrations range from 0.5% to 2% *w*/*v* and are prepared in phosphate-buffered saline (PBS) or phosphate buffer at physiological pH 7.4 [86,98,99]. The physical constraint imposed by the gel restricts swelling and promotes agglomeration of the dosage form. These are important contributing factors to the drug release kinetics that are not often encompassed in conventional (buffer) methods. For instance, Kožák et al. demonstrated that microspheres embedded in agarose gel merged into agglomerates, thereby reducing the effective surface area and slowing drug release, a phenomenon also observed in vivo but not in conventional IVR tests [99]. Agarose-based IVR systems thus could provide a more discriminative early assessment of formulation performance, enabling differentiation between formulations with varying polymer compositions, solvent systems, and device geometries that may otherwise appear similar in conventional tests [86,98,99]. The use of bromothymol blue in the gel showed acidic microenvironments at the depot surface early on, which is consistent with PLGA autocatalysis and acid accumulation reported in vivo [86]. Based on the available data, these systems appear to facilitate mechanistic investigations of processes occurring in vivo, thereby possibly enhancing the physiological relevance of the IVR test and the predictiveness of in vivo performance [86,98].

However, there are also some limitations related to agarose gel-based IVR systems. Firstly, agarose is chemically inert and lacks the complex biochemical components of the real extracellular matrix, such as proteins, hyaluronic acid, lipids, enzymes, and cells. As a result, these gels do not reproduce specific binding interactions, enzymatic degradation, or immune responses that can impact solvent exchange in the case of ISFIs, porosity, and drug–matrix interactions in vivo [99]. Secondly, although subcutaneous drug transport is primarily diffusion-driven, some degree of convection still occurs in vivo due to lymphatic flow or tissue movements. This may lead to an overestimation of diffusion barriers and a potential underestimation of drug release rates compared with the in vivo situation [98]. These factors should be considered when interpreting IVR data from agarose-based systems, as they may not fully capture the dynamic and biochemical complexity of the subcutaneous environment.

Within the context of biorelevance and drug release, significant progress has been made in the characterization of peptides and proteins. Two systems in particular have emerged in this area that may be applicable to LAI formulations: the Pion SCISSOR platform and the BioJect system.

**Figure 8:**
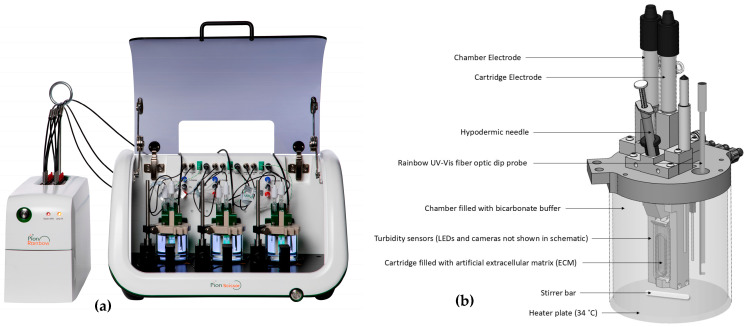
Pion Scissor system. (**a**) Pion Scissor coupled with the Pion Rainbow in situ fiber-optic UV–Vis spectrometer, (**b**) Schematic representation of the Scissor chamber. The images were kindly provided by Imogen Anastasiou, Pion Inc.

The Pion SCISSOR (Subcutaneous Injection Site Simulator) system (Figure 8), first described in 2015 and used for the testing of human insulins and monoclonal antibodies, provides a mechanistic in vitro platform to simulate the fate of biopharmaceuticals following subcutaneous injection. The system employs a dialysis-based injection chamber, which can be loaded with defined concentrations of extracellular matrix (ECM) components such as HA, enabling the study of the influence of the ECM on drug release. This chamber is immersed in a bicarbonate-based physiological buffer, maintained at 34 °C and pH 7.4, to closely mimic the ionic composition and homeostatic conditions of the subcutaneous tissue. Hydrodynamics and interstitial pressure are mimicked by controlling the fluid volume and pressure within the chamber, while a modified dialysis membrane with micropores mimics the uptake of drugs into blood and lymphatic capillaries. Real-time monitoring of pH, pressure, and drug release allows the SCISSOR system to provide valuable insights into the physicochemical transitions and interactions that govern the absorption and bioavailability of subcutaneously administered biopharmaceuticals [100].

**Figure 9:**
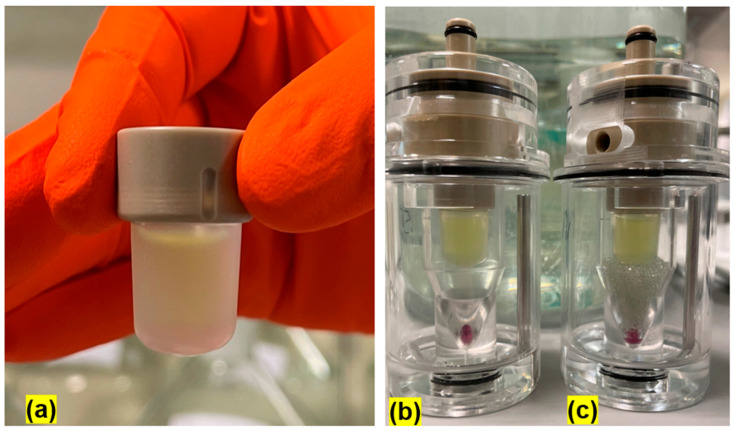
Bioject platform (by Sotax). (**a**) Bioject cell filled with yellow dye, (**b**) Bioject cell filled with yellow dye inside a flow-through cell, (**c**) Bioject cell filled with yellow dye in a flow-through cell containing glass beads.

Recently, a novel BioJect platform (Sotax) (Figure 9) was developed to closely simulate the physiological SC microenvironment [101] for different insulin formulations. It integrates a compendial flow-through cell (Apparatus 4) with a perfusion system and simulates the extracellular matrix (ECM) by incorporating a customizable biomatrix within its flow-through cell, using hydrogels such as agarose as a base and supplementing them with physiologically relevant biopolymers like collagen and hyaluronic acid. This design mimics the structural, charge, and hydration properties of the native ECM, thereby replicating tissue retention, diffusion barriers, and the dynamic interactions that influence drug release and absorption in subcutaneous tissue. The modified simulated subcutaneous interstitial fluid (mSSIF) used in this setup consisted of 138.5 mM sodium, 10 mM potassium, 1.8 mM calcium, 0.8 mM magnesium, 111.3 mM chloride, 28 mM bicarbonate, 0.5 mM sulfate, 5 mM acetate, 4.2 mM phosphate, and 30 g/L total protein (bovine serum albumin), formulated to closely mimic the ionic and protein composition of human subcutaneous interstitial fluid [101].

For more details on biorelevant subcutaneous conditions and corresponding media compositions, the reader is kindly referred to a comprehensive review by Li et al. [77].

### 5.2. Intramuscular (IM) Administration

Unlike the SC environment, muscle tissue is highly vascularized and represents a dynamic site shaped by its extracellular matrix (ECM), interstitial fluid (ISF) composition, and local blood flow, all of which influence drug fate after injection. The ECM is organized into the endomysium, perimysium, and epimysium, composed mainly of type I collagen and hyaluronic acid (HA), with proteins such as fibronectin contributing to structural strength and viscoelasticity. HA acts as a hydration reservoir and provides elasticity, supporting smooth muscle movement and reducing friction [102]. A dense capillary network enables efficient nutrient, oxygen, and drug exchange. Because muscle tissue has greater blood flow than SC tissue, intramuscularly injected drugs are typically absorbed more rapidly [102,103]. Electrolyte and protein levels are similar to those in SC tissue but fluctuate with muscle activity and perfusion changes [96]. ISF pH is around 7.4 but may vary under physiological or pathological conditions. Ultimately, the absorption and systemic availability of IM-injected drugs are dictated by the interplay of these ECM and ISF properties, as well as the physicochemical characteristics of the drug and formulation [102].

A comprehensive study by Kozak and Lamprecht [104] explored several biorelevant IVR setups using the commercial LAI Risperdal^®^ Consta^®^, including a previously developed muscle tissue-based in vitro system containing native muscle lipids [105]. Conventional in vitro testing was performed in 0.1 M phosphate buffer, pH 7.4, containing 0.02% polysorbate 20 and 0.05% sodium azide under mild horizontal agitation to represent a conventional IVR environment with freely suspended microspheres. It was demonstrated that neither the addition of enzymes nor albumin produced meaningful changes in risperidone release or PLGA degradation compared with the buffer alone. Subsequently, release media incorporating isolated muscle lipids and defined lipid components were developed to examine the role of physiological fatty acids. Media rich with fatty acids, particularly those containing oleic acid, significantly accelerated risperidone release by shortening the lag phase and inducing earlier microsphere swelling. Furthermore, an agarose gel envelope method was employed to mimic the mechanical confinement of tissue surrounding the depot after intramuscular administration. In this gel system, risperidone release was slower due to restricted swelling and merging of softened microspheres, which reduced the effective surface area available for drug diffusion. A combined agarose-lipid setup partially restored the faster early release while maintaining slower release in the later phase, improving overall biorelevance relative to either approach alone. Finally, a simulated muscle tissue setup, incorporating microspheres directly into previously freeze-dried and then rehydrated porcine muscle tissue fixed with agarose, reproduced both the biochemical lipid environment and the mechanical constraints of intramuscular tissue. This muscle-based system produced a shorter lag phase and a slower post-lag release, closely matching (published) human in vivo data. Removal of endogenous lipids from the muscle matrix stopped the early acceleration of drug release, indicating that tissue lipids drive the initial faster in vivo release. The study showed that fatty acid interactions dominate the early release phase by enhancing swelling and erosion of PLGA microspheres. It further showed that swelling restriction imposed by muscle tissue governs the later-phase slowing of release. The authors concluded that realistic IVIVR requires incorporating both biochemical (lipids) and biophysical (mechanical confinement) factors, with the simulated muscle tissue method providing the closest in vitro approximation of human in vivo release [104].

### 5.3. Intra-Articular Administration

Synovial fluid (SF) is a viscous, viscoelastic (non-Newtonian) plasma dialysate rich in HA, proteins, primarily human serum albumin, and lubricin, which supports joint lubrication and shock absorption [106,107]. In healthy joints, the synovial fluid exhibits a pH of around 7.4 and a higher viscosity (up to 1 Pa·s), higher HA levels (1.5–4 mg/mL), and lower protein content (10–30 mg/mL) compared with inflamed joints SF, where HA decreases to 0.2–1.8 mg/mL and proteins rise to about 50 mg/mL. Pathological conditions such as arthritis degrade HA and reduce viscosity, impairing lubrication and increasing joint wear and pain [106,107]. These changes in SF composition and rheology are critical for the development and evaluation of intra-articular drug delivery systems [106,107]. Joint mechanics, the high viscosity, and viscoelastic nature of SF, as well as its turnover, affect drug fate, as movement enhances mixing and may promote clearance of small particles, while disease-altered SF composition can modify drug distribution and retention [9,107]. Therefore, IVR method development for intra-articular delivery requires careful consideration of SF composition, rheology, and joint biomechanics in relation to the drug product of interest.

Magri et al. [108] employed biorelevant synovial fluid media containing HA and phosphatidylcholine with/without proteins to simulate healthy and osteoarthritic joint conditions, prepared according to Nikolettos [109]. Specifically, the healthy-state medium (H-BSF) consisted of ~8.1 mg/mL HA, 0.15 mg/mL PC, 11.5 mg/mL BSA, and 1.7 mg/mL γ-globulin [109], adjusted to pH 7.4 [108]. The osteoarthritic medium (OA-BSF) used ~4.8 mg/mL HA and 0.25 mg/mL PC, with the same BSA and γ-globulin levels [109] and a pH of 8.0 [108].

A protein-free variant, OAwp-BSF, maintained OA phospholipid and HA levels while excluding proteins, thereby enabling the isolation and study of the specific effects of proteins on drug solubility and release from PLGA microspheres and the commercial methylprednisolone acetate suspension (DepoMedrone^®^). Protein-rich BSF increased methylprednisolone (MP) solubility and release from both PLGA microspheres and a DepoMedrone^®^. Solubility and drug release were highest in OA-BSF and lowest in OAwp-BSF, indicating that albumin and γ-globulin act as solubilizing components [108].

### 5.4. Subgingival Administration

The subgingival environment presents a unique challenge for assessing biorelevance in vitro since it is characterized by complex biofilms, dynamic fluid composition, and host immune responses.

The development of biorelevant in vitro testing for subgingival administration should consider the simulation of the periodontal pocket environment. The composition of simulated saliva or gingival crevicular fluid (GCF) should include physiologically relevant concentrations of mucins (typically 1–10 mg/mL), amylase (0.5–2 mg/mL), and total proteins (0.5–3 mg/mL), as these proteins influence drug release and biofilm interactions [110,111]. The media should have a pH between 6.8 and 7.4 to reflect the microenvironment of inflamed pockets [110]. The fluid volume should be based on realistic pocket geometry; however, these volumes vary significantly among patients. In healthy subgingival sites, GCF flow rates typically range from 3 to 8 μL/h per site, with a resting volume of about 0.05–0.06 μL, while intermediate pockets exhibit flow rates around 20 μL/h, and flow rates in deep pockets can reach up to 44 μL/h or higher, with resting volumes of up to 1.5 μL. Advanced periodontitis may present flow rates as high as 137 μL/h. The rapid turnover of GCF means that the pocket volume is replaced every 1–2 min in healthy conditions and as frequently as every 15–60 s in diseased pockets [112].

An FDA-funded science grant resulted in the development of a biorelevant method using a Small Volume Apparatus (SVA), which was designed specifically to reproduce the microenvironment of the periodontal pocket by integrating physiologically relevant features [58]. This setup employed a custom-made polycarbonate chamber containing a slotted inner compartment wrapped in a 50 kDa dialysis membrane, which confined PLGA microspheres while allowing diffusion of the released drug substance into the surrounding medium. Approximately 10 mg of PLGA-based microspheres with minocycline hydrochloride (Arestin^®^ and in-house formulations) were deposited into the inner chamber and immersed in 250 µL of simulated gingival crevicular fluid (sGCF). The sGCF was prepared by dissolving citrate buffer salts, NaCl, KCl, CaCl_2_, and low-level BSA (0.05 g/L) in water and adjusting the pH to 7.25. A syringe pump was employed to flush the sGCF through the outer chamber at 0.5 µL/min. This flow rate was chosen to reflect physiological GCF turnover while ensuring sustained concentration gradients across the membrane. This design enabled IVR testing under low-volume, low-flow-rate conditions that cannot be replicated by high-volume compendial systems. Results of drug release from the SVA were compared with those of the previously developed Apparatus 4 method, which employs a higher volume and flow rate of PBS-based medium. The Apparatus 4 method produced a rapid and complete release of Arestin^®^ within 3 days, whereas the SVA method released less minocycline even over 14 days due to the very small medium volume and low flow rate that mimic periodontal pocket conditions. Although the rank order and discrimination of minocycline release were similar between Apparatus 4 and the SVA, the ability to discriminate drug release during the initial release period (<5 days) was greater for the biorelevant method. However, the formulation screening potential and QC adaptability were better with the Apparatus 4 method [58].

To mimic the constrained geometry and slow fluid turnover of the periodontal pocket, another approach of a custom-made small-volume flow-through dissolution chamber (0.06 mL) was employed [113]. The method was based on a previously developed chamber by Ren et al. [114], originally designed for gelatin matrix systems, and was adapted for PLGA-based products, including Arestin microspheres and the Atridox in situ forming implant. The chamber simulated physiological conditions through maintaining the temperature at 34 °C, providing a slow flow of simulated saliva at approximately 0.63 µL/min, and using a medium composed of simulated saliva containing 0.137 M sodium chloride, 0.0014 M potassium phosphate monobasic, 0.017 M sodium phosphate dibasic, and 0.3% trypsin, adjusted to pH 8.0. Additional modifications, including filter paper to retain Arestin microspheres within the chamber and a blocking film to restrict the exposed surface area of the Atridox in situ forming implant, were implemented to reflect in vivo constraints. Under these conditions, the methods for both PLGA products indicated biorelevance by establishing a correlation with previously published in vivo data [113].

## 6. Analysis and Comparison of IVR Profiles for PLGA-Based LAIs

Methods used for analyzing and comparing the in vitro release profiles broadly fall into two categories: model-independent methods and model-dependent methods.

Model-independent methods compare IVR profiles using calculated metrics without assuming a specific release mechanism. The most widely used model-independent method for comparing IVR profiles is the similarity factor f_2_ [115,116]. The f_2_ value is calculated based on the point-by-point difference between the two mean profiles. When the two profiles are identical, f_2_ is 100. An average difference of 10% between the two profiles at all measured time points results in an f_2_ value of 50. Calculated f_2_ values between 50 and 100 indicate similarity between profiles.

Regulatory guidelines [117,118,119] define the conditions for the use of f_2_—namely, a minimum of 12 individual values for each time point and each product, the same testing conditions and same time points for both products, a minimum of three time points, not more than one mean value of >85% and a maximum percent coefficient of variation (%CV) of 20% at early points and 10% at later points. When these conditions are met, f_2_ offers a simple way to assess the similarity of release profiles.

When an IVR dataset exhibits high variability (and the conditions for f_2_ calculation are not met), alternative model-independent approaches may be considered, such as bootstrapped f_2_ [120,121,122].

In the context of PLGA LAIs, researchers have most frequently used f_2_ for IVR profile comparison [32,52,62,63,68,84,85]. In some cases, the difference factor f_1_ [116] is also reported alongside f_2_ [52,62,68], but f_2_ remains the primary metric in IVR profile comparison.

The model-dependent approach involves fitting in vitro release data to mathematical models to evaluate the similarity of the dissolution profiles and to help understand the drug release mechanism. Several PLGA-based LAI studies have applied model-dependent methods to describe the release kinetics and to support qualitative interpretation of the underlying release mechanisms. Commonly used models include zero-order, first-order, Higuchi, Weibull, and the Korsmeyer–Peppas model (including its two-phase extension, Peppas–Sahlin). Multiple models are usually evaluated in parallel: for example, Zhang and Fassihi fitted six standard kinetic models to naltrexone PLGA ISFI in vitro data and found that the Peppas–Sahlin model provided the best fit, consistent with a predominantly diffusion-controlled release mechanism [62]. In another case, Wang et al. applied first-order and Higuchi models to assess in vitro and in vivo release and in vitro degradation kinetics of risperidone ISFIs. They reported that in vivo profiles were best fitted by the Higuchi model, while the in vitro degradation-facilitated release followed first-order kinetics [123].

To conclude, f_2_ is widely used as the primary model-independent method for IVR profile comparisons of PLGA LAI formulations. At the same time, a variety of model-dependent methods have been applied to analyze the IVR profiles and to gain insight into the underlying release mechanism. It should be emphasized, however, that mathematical modeling should be used with caution when drawing conclusions about the release mechanisms, as the goodness-of-fit alone does not establish the true release mechanism [124].

## 7. In Vitro-In Vivo Correlation

In vitro-in vivo correlation (IVIVC) is defined as a mathematical relationship between an in vitro property of a dosage form, usually drug release kinetics, and a relevant in vivo response [125,126]. A Level A IVIVC is the most informative category of IVIVC because it represents a point-to-point relationship in which the entire in vivo absorption profile or in vivo dissolution profile can be predicted from the in vitro release profile. Although Level A IVIVC is required for biowaivers, lower-level IVIVCs may still support regulatory decision-making when they are scientifically justified, validated, and fit for purpose [91,125,126]. The term in vitro–in vivo relationship (IVIVR) is also used to describe semiquantitative or rank order relationships between in vitro release and in vivo outcomes [81].

IVIVC can be used to set clinically relevant dissolution specifications. This implies the establishment of a link between drug product quality attributes (e.g., in vitro release), critical material attributes (CMAs), critical process parameters (CPPs), and in vivo performance (e.g., systemic exposure) [127].

For PLGA-based LAIs, developing IVIVCs is particularly challenging because in vivo release depends on processes such as tissue hydration, enzymatic activity, polymer degradation, and dynamic changes in depot morphology, factors that cannot be fully replicated in standard in vitro tests. In addition, the long release duration of PLGA systems complicates the alignment of in vitro and in vivo data [76]. A comprehensive discussion of these challenges and methodological considerations in the development of IVIVC for PLGA-based LAIs, with a primary focus on microsphere formulations, is provided in Wang et al. (2025), to which the reader is kindly referred for further guidance [81].

Experience with IVIVC development for PLGA-based ISFIs has been more limited than for microspheres, reflecting the intrinsic complexity of these systems. Following injection, ISFIs form depots in vivo through solvent exchange and polymer precipitation, leading to time-dependent changes in depot geometry, surface-to-volume ratio, and microstructure, with the drug substance, solvent, and polymer each playing distinct roles. Recent studies have shown progress toward IVIVC for ISFIs. Yang et al. reported a level A IVIVC for PLGA ISFIs loaded with eprinomectin in a rat subcutaneous model by using a tubule Sample and Separate in vitro release method. By evaluating formulations with different PLGA molecular weights and varying ratios of fast- to slow-release solvents (NMP to glyceryl triacetate), this study showed that the IVR method was discriminatory and able to predict the in vivo performance of the tested ISFIs [38]. In another study, the Burgess group reported a Level A IVIVC in a rabbit model for risperidone ISFIs with varying PLGA molecular weight (while keeping the polymer’s lactic/glycolic ratio and end-cap chemistry constant). IVIVC was achieved using an in vitro method with Apparatus 2 and a glass slide adaptor, and it was found that the surface-to-volume ratio and water uptake ratio were the most critical method attributes that should be carefully controlled. The authors reported successful external validation, whereas prediction accuracy for peak metrics (Cmax) was more challenging to achieve [71]. Overall, available studies suggest that IVIVC for ISFIs can be achieved under carefully controlled conditions; however, it remains challenging and highly dependent on the experimental approach and formulation characteristics, underscoring the need for further studies in this field.

## 8. Towards Closing the Gap in Regulatory Guidance Related to IVR Testing of PLGA-Based LAIs

The total body of research related to PLGA LAIs is immense, and the collected knowledge related to mechanisms of drug release and IVR methods for PLGA-based systems has significantly expanded over the last couple of decades.

The regulatory agencies share a generally aligned, science-based approach to in vitro release testing of LAIs. Namely, the IVR methods should be discriminatory, the IVR specifications should be based on the performance of clinical/bioavailability batches, and the establishment of IVIVC is advisable [15,91,125,126].

However, there are still no standardized compendial IVR methods for the assessment of LAIs. In fact, it has been noted that one of the main challenges associated with the development of generic PLGA-based LAIs is the lack of compendial IVR methods for LAIs [128]. Considering the complexity of drug release from PLGA-based delivery systems, a close collaboration between regulatory agencies, industry, and academia is needed in order to overcome this challenge.

The U.S. FDA launched a research program for PLGA-based drug products in 2013, supported by funding from the Generic Drug User Fee Amendments (GDUFA). Within this program, the FDA awards research grants and contracts to address the scientific gaps related to PLGA-based generic drug products. The program encompasses the following research areas: (1) analytical methods for characterization of PLGA polymers and PLGA-based formulations; (2) IVR testing methods and IVIVC for PLGA-based drug products; (3) the impact of the variations in raw materials and manufacturing process on drug release and drug-polymer interactions; and (4) modeling tools to facilitate formulation design and bioequivalence study design for PLGA-based formulations [128].

The results of the FDA research program for PLGA-based drug products have been communicated through peer-reviewed publications, posters, and presentations in scientific conferences and workshops, as well as in annual GDUFA Science and Research Reports [129]. Furthermore, the outcomes of the FDA research program also support the publication of FDA Product-Specific Guidances (PSGs) for PLGA-based drug products.

Publications produced through the FDA research program cover various topics related to IVR methods for PLGA LAIs, including the development of accelerated IVR methods for PLGA microspheres [34,41], evaluation of the impact of IVR testing conditions on in vitro release kinetics [27], development of biorelevant IVR methods [58], evaluation of the impact of PLGA attributes on the performance of ISFIs [19,123], development of IVIVC for PLGA microspheres [31,37,44,93] and investigation of in vitro and in vivo drug release mechanisms from PLGA microspheres [11,28,32,78]. The research advancements gained through this program are certainly expected to facilitate the development of discriminatory and clinically relevant in vitro methods for PLGA-based LAIs.

## 9. Conclusions and Future Considerations

Development of in vitro release methods for PLGA-based LAI products is challenging due to the lack of standardized methods, the diversity of these drug delivery systems, and the complexity of the involved drug release mechanisms. Various in vitro release methods have been explored, which can broadly be grouped into sample and separate methods, dialysis methods, and flow-through methods, each of them with its own advantages and shortcomings. IVR method development requires a careful assessment of experimental parameters, including apparatus selection, medium composition, temperature, and agitation, with controlled depot formation being particularly critical for in situ forming implants.

During early drug product development, biorelevant IVR methods, which closely mimic the physiological conditions at the administration site, may be a valuable tool for formulation development and optimization. However, the complexity of these methods frequently limits their use for routine quality control. The development of QC methods often involves striking an optimal balance between biorelevance and practicality. QC methods should be simple, robust, and reproducible while demonstrating adequate discriminatory power towards relevant critical material attributes, critical formulation variables, and/or critical process parameters. Accelerated methods may be justified, provided they maintain the same release mechanism (or at least the same rank order between formulations) when compared with the real-time method. Ultimately, the clinical relevance of the final method is desirable, implying that a link has been established between the in vitro release and in vivo performance.

One of the main challenges associated with the development of generic PLGA-based LAIs is the lack of compendial in vitro release methods that would be sufficiently discriminatory and capable of predicting in vivo performance [128]. There is, therefore, a strong need for additional regulatory guidance in this field. In this regard, the efforts made by the regulatory agencies to address the scientific gaps related to IVR testing of LAIs (such as the U.S. FDA GDUFA research program) are certainly encouraging and are expected to facilitate both the development and regulatory approval of PLGA-based LAIs.

Future progress in IVR methods for PLGA-based LAIs will likely be related to a deeper mechanistic understanding of the involved release mechanisms both in vitro and in vivo, as well as a deeper understanding of the complex and dynamic physiological conditions present at the intended administration sites, enabled by the adoption of emerging technologies, such as in situ analytical monitoring and advanced computational modeling.

## Figures and Tables

**Table 1:** In vitro release methods used for PLGA LAI products according to the USP Dissolution Methods Database [20].

Monograph	Apparatus	Medium	Volume (mL)	Test Time	Exceptions	Description of the Drug Delivery System ^1^
Goserelin Implants	Flat-bottom glass jar	Phosphate/citrate buffer, pH 7.4	50	168, 336, 408, 504, 672 h	39 ± 0.5 C	Extended-release PLGA-based implants (3.6 mg) for s.c. administration [20,24]
Goserelin Implants	Flat-bottom glass jar	Phosphate-buffered saline, pH 7.4	50	72, 336, 840, 1344, 2016 h	39 ± 0.5 C	Extended-release PLGA-based implants (10.8 mg) for s.c. administration [20,24]
Minocycline Periodontal System	Tube rotator	Phosphate buffer, pH 4.2	10	4, 24, 48, 72 h	catalog # 400110 Labquake	Sustained-release PLGA-based microspheres for subgingival administration into periodontal pockets [20,25,26]

^1^ The data presented in column Description of the drug delivery system in this table has been compiled by the authors based on information available in USP Dissolution Methods Database [20], USP drug product monographs [24], FDA Orange Book Database [25] and Drugs@FDA Database [26].

**Table 2:** In vitro release methods used for PLGA LAI products according to the FDA Dissolution Methods Database [21].

Drug Name	Dosage Form	Apparatus	Speed (rpm)	Medium	Volume (mL)	Recommended Sampling Times	Description of the Drug Delivery System ^1^
Dexamethasone	Implant (intravitreal)	VII (with reciprocating 50 mesh baskets)	30 cycles per min	Phosphate-buffered saline containing 0.05 g/L sodium dodecyl sulfate at 45 ± 0.5 °C	30	12, 24, 48, 72, 96, 120, 144, 168, 192, 216, and 240 h	Sustained-release PLGA-based intravitreal implant, for intravitreal injection [21,25,26]
Goserelin acetate	Implant	120 mL Wheaton jar	Swirl orbit of 50 mm at 205 rpm for 6 s (Prior to sampling, the jar is removed from incubation and mechanically swirled with a digital orbital shaker)	50 mL of phosphate-buffered saline, pH 7.4, at 39 °C (warmed overnight before the implants are added)	50	3, 14, 35, 56, and 84 days (10.8 mg strength); 7, 14, 17, 21, and 28 days (3.6 mg strength)	Sustained-release PLGA-based implants for s.c. administration [21,25,26]
Naltrexone	Injectable suspension	Develop an in vitro release method using Apparatus IV (flow-through cell), and, if applicable, Apparatus II (paddle) or any other appropriate method, for comparative evaluation by the Agency	/	Phosphate-buffered saline with 0.02% Tween 20 and 0.02% Sodium azide, pH 7.4 (final osmolality should be 270 ± 20 mOsm), or any other appropriate medium, at 37 °C.	/	/	Extended-release injectable suspension for i.m. administration, containing PLGA microspheres [21,25,26]
Risperidone	For suspension (extended release)	IV (Flow through cell-closed loop)	/	50 mM potassium phosphate buffer, pH 7.4	1000	8, 24, 96, 120, 144, 168, 192, 216, and 264 h	Extended-release injectable suspension for i.m. administration, PLGA-based in situ microimplant [21,25,26]
Triamcinolone Acetonide	Intra-articular, for suspension (extended release)	II (paddle)	75	0.3% SDS in 10 mM phosphate buffer, pH 7.2 + 0.02% sodium azide at 35 °C	1000	1, 2, 4, 8, 12, 16, 24, 36, 48, 72, 96, and 120 h	Extended release injectable suspension for i.a. administration, containing PLGA microspheres [21,25,26]
Triptorelin Pamoate	For intramuscular suspension (extended release)	II (Paddle)	75	50 mL of methanol to 950 mL of water	950	1, 8, 24, 96, and 168 h	Extended-release injectable suspension for i.m. administration, containing PLGA-based microgranules [21,25,26]
Triptorelin Pamoate	Injectable suspension	II (Paddle)	200	Water-Methanol (95:5); Reconstitute vial in 2 mL of Water for Injection, add to 500 mL medium at 37 °C	500	1, 6, 12, 24, 48, and 72 h	Depot injectable suspension for i.m. administration, containing PLGA-based microgranules [21,25,26]

^1^ The data presented in column “Description of the drug delivery system” in this table has been compiled by the authors based on information available in FDA Dissolution Methods Database [21], FDA Orange Book Database [25] and Drugs@FDA Database [26].

**Table 3:** Potential advantages and disadvantages of the sample and separate, flow-through and dialysis methods.

IVR Method	Advantages	Disadvantages
Sample and separate	Simple and widely available equipment Easy to set up Cost-friendly Flexibility of medium volume Flexibility of equipment selection Suitability for long-term studies	Floating of microparticles Aggregation of microparticles Cumbersome sampling technique (in case of microparticles) Unintended sample loss during sampling Limited inter-laboratory comparability (in case of non-standardized equipment)
Flow-through	Standardized pharmacopoeial apparatus Convenient sampling and medium replacement Physical separation of the microparticles from the bulk medium Flexibility of medium volume Potential automation Potential coupling with fiber-optic UV probes	Complexity of the apparatus Expensive equipment Cumbersome setup procedure Filter blockage Failure of O-rings or filters during prolonged testing Potential adsorption of analyte onto hydrophobic surfaces
Dialysis method	Convenient sampling and medium replacement Physical separation of the microparticles from the bulk medium	Limited inter-laboratory comparability (in case of non-standardized equipment) Cumbersome setup procedure (in case of dialysis bags) Lack of agitation in the donor compartment Violation of sink conditions in the donor compartment Slow equilibration between donor and acceptor compartments Binding of the analyte to the dialysis membrane

**Table 4:** Key implant formation factors and their impact on IVR of PLGA-based ISFIs.

Implant Formation Factor	Experimental Control/Setup	Effect on Depot Geometry and Microstructure	Impact on IVR Profile	References
Implant formation method (free injection vs. geometry-controlled formation)	Free injection into the release medium vs. formation within molds or holding cells	Free injection produces irregular shapes and sizes. Geometry-controlled formation yields uniform depots with defined dimensions.	Irregular depots produced by the free injection method exhibit poor IVR reproducibility. Geometry-controlled depots show low variability and consistent IVR profiles.	[19,54,62,67,71,74]
Injection volume	Comparing small injection volumes (e.g., ~50 µL) vs. full dose (e.g., 250 µL) in a free injection setup	Small volumes tend to form compact, spherical, and uniform depots. Larger volumes increase shape variability (with free injection setup).	Larger injection volumes are associated with a higher initial burst and poorer reproducibility under free-injection conditions.	[67]
Mechanical confinement during formation	Unconfined formation vs. confined formation (e.g., dialysis bags, adapters with an insoluble membrane, or glass slides)	Unconfined depots may overly swell, deform, or fragment. Confinement stabilizes implant shape and prevents loss of fragments during testing.	Confinement markedly lowers the initial burst and improves reproducibility, owing to the constant depot geometry during testing.	[19,67,71]
Surface to volume ratio (S/V)	Implants with varying S/V ratios obtained by various formation methods (free injection, dialysis bag, PVA thin film, gelatin capsule, sandblasted glass slide, etc.)	Higher S/V (thinner depots) enables faster phase inversion. Lower S/V yields thicker depots with longer diffusion distances and consequently slower phase inversion.	Higher surface area depots exhibit higher initial burst release with shorter lag phases. Lower surface area depots tend to have lower initial burst release and exhibit pronounced lag phases.	[19,67,71]
Rate of phase inversion (solvent–water exchange)	Use of a diffusion barrier or adapter vs. no barrier; measurement via solvent release	Slower phase inversion allows implants to solidify more evenly. Rapid phase inversion creates a porous, less dense matrix.	Slower phase inversion tends to decrease the initial burst release.	[54,67,71,75]

**Table 5:** Studies investigating the elevated temperature approach for accelerated IVR testing from PLGA-based drug delivery systems.

Drug Substance	Type of Drug Delivery System	Experimental Setup	Temperature	Reference
Dexamethasone	PLGA microparticles	FT	45, 53, 60 and 70 °C	[83]
Leuprolide	PLGA microparticles	DM	50, 55, 60 °C	[60]
Leuprolide	PLGA microparticles	DM	55 °C	[55]
Naltrexone	PLGA microparticles	SS, FT	45 °C	[34]
Risperidone	PLGA microparticles	SS, FT	45 °C	[41]
Risperidone	PLGA microparticles	SS, FT	45 °C	[27]
Risperidone	PLGA microparticles	FT	45 °C	[52]
Risperidone	PLGA microparticles	FT	45, 50, 54.5 °C	[82]
Risperidone	PLGA microparticles	FT	45 °C	[53]
Risperidone	PLGA microparticles	SS	45, 50, 55 °C	[84]
Triamcinolone acetonide	PLGA microparticles	FT	39 °C	[79]
Thymopentin	PLGA microparticles	SS	40, 45, 50, 55 °C Temperature gradient (40 => 45 => 50 °C)	[85]
Leuprolide	PLGA ISFI	Agarose gel-based IVR setup	50, 55, 60 °C	[86]
Risperidone	PLGA ISFI	SS + ISFI adapter	40 °C	[54]

FT = flow-through method; DM = dialysis method; SS = sample and separate method.

## Data Availability

No new data were created or analyzed in this study.
